# An Efficient Brain Tumor Segmentation Method Based on Adaptive Moving Self-Organizing Map and Fuzzy K-Mean Clustering

**DOI:** 10.3390/s23187816

**Published:** 2023-09-12

**Authors:** Surjeet Dalal, Umesh Kumar Lilhore, Poongodi Manoharan, Uma Rani, Fadl Dahan, Fahima Hajjej, Ismail Keshta, Ashish Sharma, Sarita Simaiya, Kaamran Raahemifar

**Affiliations:** 1 Department of Computer Science and Engineering, Amity University Gurugram, Gurugram 122412, Haryana, India; 2 Department of Computer Science and Engineering, Chandigarh University, Mohali 140413, Punjab, India; 3 College of Science and Engineering, Hamad Bin Khalifa University, Qatar Foundation, Doha P.O. Box 5825, Qatar; 4 Department of Computer Science and Engineering, World College of Technology & Management, Gurugram 122413, Haryana, India; 5 Department of Management Information Systems, College of Business Administration Hawtat Bani Tamim, Prince Sattam Bin Abdulaziz University, Al-Kharj 11942, Saudi Arabia; 6 Department of Information Systems, College of Computer and Information Sciences, Princess Nourah Bint Abdulrahman University, Riyadh 11671, Saudi Arabia; 7 Computer Science and Information Systems Department, College of Applied Sciences, AlMaarefa University, Riyadh 13713, Saudi Arabia; 8 Department of Computer Engineering and Applications, GLA University, Mathura 281406, Uttar Pradesh, India; 9 Apex Institute of Technology (CSE), Chandigarh University, Gharuan, Mohali 140413, Punjab, India; 10 Data Science and Artificial Intelligence Program, College of Information Sciences and Technology, Penn State University, State College, PS 16801, USA; 11 School of Optometry and Vision Science, Faculty of Science, University of Waterloo, 200 University, Waterloo, ON N2L 3G1, Canada; 12 Faculty of Engineering, University of Waterloo, 200 University Ave. W., Waterloo, ON N2L 3G1, Canada

**Keywords:** brain tumor, adaptive self-organizing map, K-means, gray level co gray level co-occurrence matrix, medical imaging

## Abstract

Brain tumors in Magnetic resonance image segmentation is challenging research. With the advent of a new era and research into machine learning, tumor detection and segmentation generated significant interest in the research world. This research presents an efficient tumor detection and segmentation technique using an adaptive moving self-organizing map and Fuzzyk-mean clustering (AMSOM-FKM). The proposed method mainly focused on tumor segmentation using extraction of the tumor region. AMSOM is an artificial neural technique whose training is unsupervised. This research utilized the online Kaggle Brats-18 brain tumor dataset. This dataset consisted of 1691 images. The dataset was partitioned into 70% training, 20% testing, and 10% validation. The proposed model was based on various phases: (a) removal of noise, (b) selection of feature attributes, (c) image classification, and (d) tumor segmentation. At first, the MR images were normalized using the Wiener filtering method, and the Gray level co-occurrences matrix (GLCM) was used to extract the relevant feature attributes. The tumor images were separated from non-tumor images using the AMSOM classification approach. At last, the FKM was used to distinguish the tumor region from the surrounding tissue. The proposed AMSOM-FKM technique and existing methods, i.e., Fuzzy-C-means and K-mean (FMFCM), hybrid self-organization mapping-FKM, were implemented over MATLAB and compared based on comparison parameters, i.e., sensitivity, precision, accuracy, and similarity index values. The proposed technique achieved more than 10% better results than existing methods.

## 1. Introduction

Medical significance dramatically influenced many researchers with the development of image-processing technologies. Today’s imaging techniques include computerized tomography scans, X-rays, positron emissions tomography (PET), and magnetic resonance imaging (MRI). MRI is the most frequently used diagnosis of brain tumors [1]. The doctors decide on therapy by assessing the present condition of the tumor based on the diagnostic value reported. Doctors devise treatments depending on the significance stated in the diagnosis process by analyzing the current situation of the tumor [2].

The tumor treatment depends on the tumor’s nature and size. However, both the form and location are most important. It is necessary to recognize the tumor as benign or non-benign. In the brain with irregular tissues, the tumor can cause uncontrollable growth. The non-benign tumor can be appropriately removed without affecting any natural tissues and redeveloped [3]. Non-benign tumors are sometimes considered malignant tumors that control the role of the neighborhood cells of the brain. This happens primarily because of the excessive development of irregular tissues. A benign tumor [4] is a distinctive array of tissues in the brain. It does not depend on the age of humans and occurs in any malefic. This task can be carried out with various methods, such as radiotherapy or chemotherapy.

**Figure 1:**
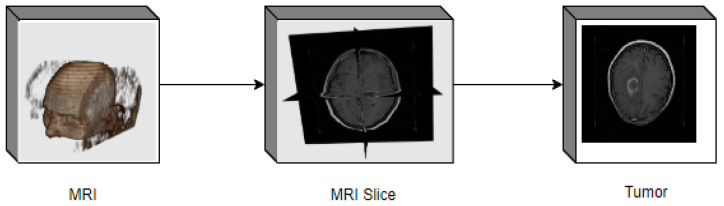
MRI acquisition for brain tumor detection.

The critical distinction is that benign cancers are homogenous, while malignant tumors are homogenous. Their specific features vary because benign tumors [5] are chemotherapy/radiation therapies that radiological operatively pulverize malignant tumors. The MRI technique offers detailed brain knowledge for successfully treating brain tumors, so any tumor region can be quickly identified in Figure 1. Trained experts utilize magnetic resonance imaging (MRI) in qualitative and quantitative analysis that depends on human vision. This is limited to eight bits of grey color as visual checks. The doctors assess brain tumor prevalence in grades I, II, III, and IV.

In Figure 1, the three Tesla Siemens Magnetom Spectra MR system was used to collect these test pictures. Then, the images were converted into MRI slices. The brain has a complex body system closely linked to the skull. Segments of neurologists note that MRI is carried out manually by slice selection. However, it is time intensive, distracting, and leads to an incorrect diagnosis. The main goal of medical imaging is to obtain meaningful and accurate information from these images with as little error as possible. Of the various medical imaging methods available to us, MRI is the most reliable and safest. The body is not exposed to any harmful radiation. The proposed work implements an energy-efficient and hybrid segmentation approach to minimize this problem.

It uses a self-organizing adaptive map and k-means to execute [6]. Segmentation is intended to distinguish between the brain tumor area from the pathological one and the brain tumor from the normal one. The procedure uses the hybrid GLCM algorithm to segment tumors efficiently. A modern technique is used to measure the amount of tumor after segmentation. Traditional methods make identifying a brain tumor from an MRI image challenging. The significant advancements for locating brain tumors are image enhancement and identification. Different techniques include GLCM, statistical, texture, region-based, and wavelet features [7].

Additionally, various ways exist to remove the necessary elements from the picture. Utilizing a wide variety of characteristics for classification acts as a strong deterrent. Generally, processing the images while segmenting the relevant region from the whole frame is challenging. Image segmentation splits an entire image into many sets with similar characteristics for different areas [8]. This method involves the amount of grey background or colour, form, shape, contrast, rarity, and luminosity. This research presents brain tumor segmentation using a hybrid model. The proposed hybrid model utilizes an adaptive method that augments an ASOM with Fuzzy K-means cluster formation. The critical contribution of this research is as follows:

- This research aims to suggest an automated workflow that can automatically accurately identify and classify brain tumors. The proposed model’s initial training images were compiled using the GLCM feature extraction method. One of the most well-known feature extraction techniques is GLCM, which can determine the textural connection among an image’s pixels;
- This research utilizes the online Kaggle brain tumor dataset;
- An FKM is used to distinguish the tumor region from the surrounding tissue;
- The proposed AMSOM-FKM technique and existing methods, i.e., Fuzzy-C-means and K-mean (FMFCM), hybrid self-organization mapping-FKM, were implemented over MATLAB and compared based on comparison parameters, i.e., sensitivity, precision, accuracy, and similarity index values;
- The proposed model achieves better precision, accuracy, and sensitivity than existing methods.

The complete research is organized as follows: Section 2 covers the related work, Section 3.1 covers the dataset details, Section 3.2 explains the proposed method, Section 4 covers the experimental results and analysis, and Section 5 covers the conclusion and future works.

## 2. Related Work

An MRI scan automation system using ML was presented in research [1] for detecting brain cancers. The suggested system went through three phases of implementation. The first step involved determining if the MR scans showed any signs of malignancy (binary approach). Second, using a multi-class method, MR scans were analyzed to identify four distinct tumor types, i.e., normal, glioma, meningioma, and pituitary. Finally, CAMs of each tumor kind were developed as a supplementary resource for the specialists’ tumor identification efforts. The findings of the ResNet50, InceptionV3, and MobileNet designs indicated a 100% overall accuracy for the binary technique. At the same time, for the VGG19 architecture, the figure was 99.71%. A brain tumor automation message was presented in [2]. The main emphasis of this research was on detection, localization, and segmentation. Using test data from 793 brain tumors, a 2-D superpixel segmentation method was used to accurately segment the tumor, with an average dice index of 94 ± 2.6%. The suggested approach was used to MRI images from the BraTS2018 Dataset to demonstrate its efficacy. The proposed methodology’s strength and clinical relevance were shown by comparing its performance assessment parameters to those of the gold standard approach.

An OHDNN (automatically optimized hybrid deep neural network)-based method for detecting brain tumors was presented in [3]. The suggested method comprised two parts. Once the data are assembled into pictures, they undergo pre-processing procedures, including image enhancement and noise reduction. Next, the photos go through a categorization procedure after being cleaned up. In this research, OHDNN was employed for the classification procedure. In addition, the adaptive rider optimization (ARO) technique was used to arbitrarily choose a parameter from among those available in the classifiers to boost the Convolutional Neural Network- Long Short-Term Memory Networks (CNN-LSTM) classifier’s performance. We used an MRI image dataset in our experiments.

Brain tumor segmentation using CT was presented in [4]. Traumatic brain injuries, malignant tumors, and skull fractures can all be diagnosed using CT scans. We extracted pictures from the brain tumor database as a test subject in this research project. Images were cleaned of noise and high-frequency artefacts in the pre-processing phase. Median filters are special nonlinear digital filters that reduce unwanted background noise in digital images and signals. The proposed method employed a genetic algorithm (GA) in conjunction with centroid improvements such as grey wolf optimization (GWO) and social spider optimization (SSO) to boost the precision of the FCM centroid. Compared to prior studies, the one recommended here achieved the highest possible execution in a cancer picture segmentation assessment. The results showed that when compared to using separate algorithms, the hybrid approach (SSO-GA) yielded the best accuracy (99.24%). This study used MATLAB 2014 to create a classification and segmentation method for brain tumors.

A lightweight implementation of U-Net was presented in [5]. The suggested architecture provided real-time MRI image segmentation. It provided this without requiring much data to train the proposed lightweight U-Net. Furthermore, no extra data augmentation process was needed. As a bonus, this study illustrated how the three perspective planes might be used in place. The ResNet50 network was used to identify brain tumors, as presented in [6]. They analyzed the results using a variety of traditional data augmentation approaches. We also provided our main component analysis-based technique. The ImageNet Dataset was used for training with a network learned from zero and transferred learning. Through this study, we increased our F1 detection rate to 92.34%. Using the recommended strategy and implementing learning transfer, we obtained this score using the ResNet50 network. Additionally, it was also determined, using the Kruskal–Wallis test statistic, that the suggested approach is distinct from the other traditional methods at the 0.05 level of significance.

Intracranial tumor segmentation (ICTS) data were constructed and presented in [7]. This data set was compiled from actual hospital radiosurgery procedures and shaped by experienced neurosurgeons and radiation oncologists. It included T1-weighted pictures with contrast added from 1500 patients and labelled which tumors needed to be removed. Artificial intelligence (AI)-based categorization of brain cancers using convolution neural network (CNN) methods was presented in [8] for use with publicly available datasets for this purpose. It can identify (also known as categorize) tissue as a tumor or non-neoplastic. Super-resolution approach and ResNet50 architecture detect an accuracy rate of 98.14% inside the framework.

A contactless tumor removal system to remove phantom tumor tissue autonomously was presented in [9]. When the size of the internal tumor varied from 7.5 to 12.5 mm, there was no change in the system’s functioning. The categorization of brain tumor pictures without human interaction was presented in [10], in which several standards and hybrid ML models were constructed and evaluated in depth. These models were considered to find the most effective model for using neural networks for brain tumor classification. Finally, a stacked classifier was presented that used several distinct cutting-edge methods to surpass the others. Their performance was 99.2%, 99.1%, and 99.2%, respectively.

A convolutional layer to execute a convolution operation for segmenting and recognizing MRI brain tumors was presented in [11]. Several classification algorithms were used to determine if an image was normal or pathological. After the data were classified, the Fuzzy C-Means (FCM) clustering method and its related optimization approaches were used to keep tabs on the aberrant photos and to choose which ones to segment. In [12], models were used to compare their performance in detecting and categorizing two distinct brain tumors. The characteristics needed for brain tumor classification were first retrieved from several Inception modules. Using Inception-v3 and DensNet201 on test samples, the suggested technique obtained the maximum performance in detecting brain tumors, with testing accuracies of 99.34% and 99.51%, respectively, as demonstrated by the findings. This work suggested strategy is based on the concatenation of features.

An approach for image compression using a deep wavelet autoencoder (DWA) was presented in [13]. When used together, they dramatically reduced the feature set size that needs to persist through a subsequent classification task using DNN. It was evaluated on a dataset consisting of brain images. When comparing the DWA-DNN classifier’s performance criterion to those of other classifiers, it was found that the suggested technique excelled. Using machine learning techniques was presented in [14]. It offered a noninvasive automated diagnostic approach for gliomas. First, standard pictures were produced using image standardization techniques such as size normalization and background removal; next, low-contrast traditional brain images were improved via the modified dynamic histogram equalization; last, skull removal via outlier identification was provided.

A multi-CNNs technique to identify brain cancers, combining multimodal information fusion with convolution neural networks, was presented in [15]. First, this research utilized multimodal 3D-CNNs, an extension of the 2D-CNNs, to produce brain lesions with varying modal features over three dimensions. As a result, it could better extract the modal of the differences in information and solve the problem of the comprehensive neighbourhood of faults needed by 2D-CNNs for their raw input. Once the network’s convergence speeds are optimized by adding a genuine normalization layer between the convolution layers and the pooling layer, the overfitting issue may be addressed. The testing findings demonstrated that the suggested approach for detecting brain tumors could accurately pinpoint tumor lesions with improved correlation coefficient, sensitivity, and specificity outcomes. The detection accuracy was much higher than it.

A Sobel edge operator using a closed-contour algorithm with an image-dependent threshold method was presented in [16]. In another piece of research [17], using the multi-threshold K-means algorithm, a CAD (computer-aided design) machine method was used to detect the tumor area and shape. A study [18] reviewed numerous methods for diagnosing neoplasm. It also developed a hybrid approach for classifying brain tumors from MRI images. It introduced various techniques of classification. Research [19] reported the automated morphological identification and differentiation of non-enhancing tumors from stable brain tumors by localization processes. It implemented an automatic CNN segmentation method that considered all local features, an input image, and global area features—developed a fully automated brain tissue detection system using fluid-attenuated investment recovery image MRI images.

A noise reduction technique [20] that could remove special Image features was used in a study. A hybrid approach to discrete wavelet transformation was discussed in [21] to differentiate ordinary or irregular photographs of MRI brain tumors. A revolutionary strategy of classification that would take the possible vector quantization of normal brain tissue segment-damaged segment was also discussed. A new, improved approach with feature optimization for detecting brain tumors was used in [22]. Their scheme used a threshold algorithm and the comparative brain identification test to increase accuracy and decrease difficulty during medical image segmentation. The soft edge detection operator performed the image boundary extraction. A robust, intelligent, creative algorithm method that reduces the impact of image endorsing and blurring was discussed in [23]. This approach removed the threshold-based MRI brain tumor portion. Morphological procedures were used to assess edge limits and remove brain skulls correctly. The previous optimization methods were stuck with optimal local stages, but certain implementations crashed, so PSO lacked certain features [24].

To solve this problem, [25] introduced SOM-FKM for segmentation and classification on MRI images but faced the problem of area overlap. It also introduced KMFCM and intensity adjustment by analyzing volume. The threshold in Alzheimer’s disease was used by [26] to incorporate current and enhanced segmentation methods for MRI segmentation. Nearly all of the methodologies mentioned earlier rely upon segmentation of the MR brain image sequence, while the proposed approach promoted the clear distinction of the T1, T2, and FLAIR image sequences [27]. Table 1 shows the summary of existing works below.

## 3. Materials and Methods

In every clinical evaluation technique, the overall performance of the evolved analysis device depends on the database taken into consideration based on the trouble to be solved.

### 3.1. Dataset

**Figure 2:**
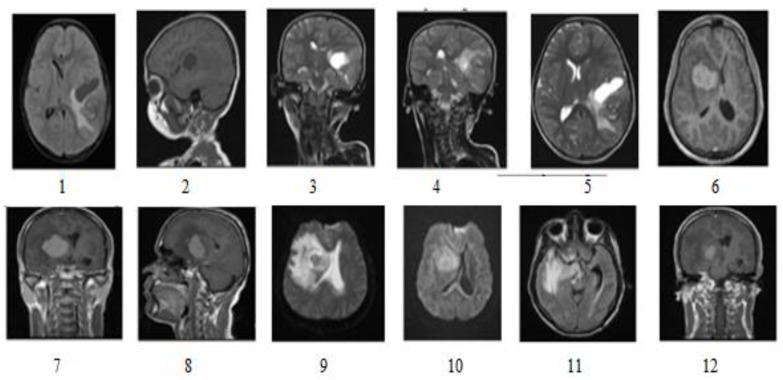
Dataset Includes different types of image sequences 1–12 (T1, T2, and FLAIR) [22].

The proposed model utilized the online KaggleBraTS 2018 dataset [27]. A single image session with visualization parameters of MRI scanners included (Siemens, Erlangen, Germany): Repetition Time T.R. = 9.8 ms, Echo Time T.E. = 4.0 ms, Rotating angle = 10, Inversion Recovery Time T.I. = 20 ms, Delay Time T.D. = 200 ms, 128 sagittal 1.25 mm gapless slices, and 256: 256 (1 × 1 mm) pixel size in the image dataset [28]. The clinical tumor case between 21 and 37 was used with 1696 images, 12 in Figure 2. Brain imaging samples of MR (magnetic resonance imaging) were classified as images of T1, T2, FLAIR (fluid-attenuated reversal recovery), and MRS (magnetic resonance spectroscopy).

**Figure 3:**
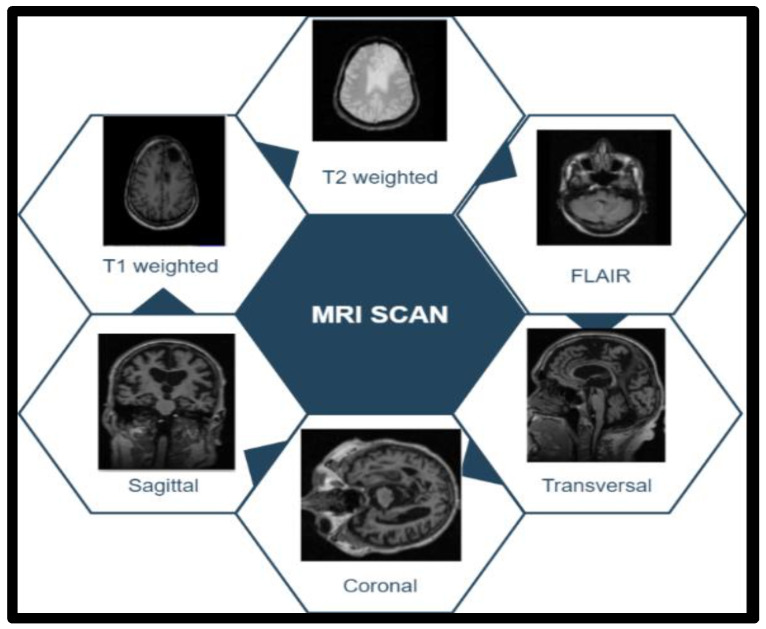
Tumor image categories in the dataset.

T1 images represent white matter in white (internal tissue area) and grey matter (external tissue area) in grey [28]. In the case of T2-weighted images, it is white for grey matter and grey for white matter. FLAIR visualization allowed the radiologist to properly visualize the brain tissue by removing the brain’s fluid material (water and brain fluid) through sagittal, coronal, and transverse MRI scan tranches. Figure 3 shows tumor image categories in a dataset. The training in set included 70% of the total images, the validation set contained 20%, and the testing set included 10%. The picture sequences T1, T2, and FLAIR were used for the proposed function.

### 3.2. Proposed Method

**Figure 4:**
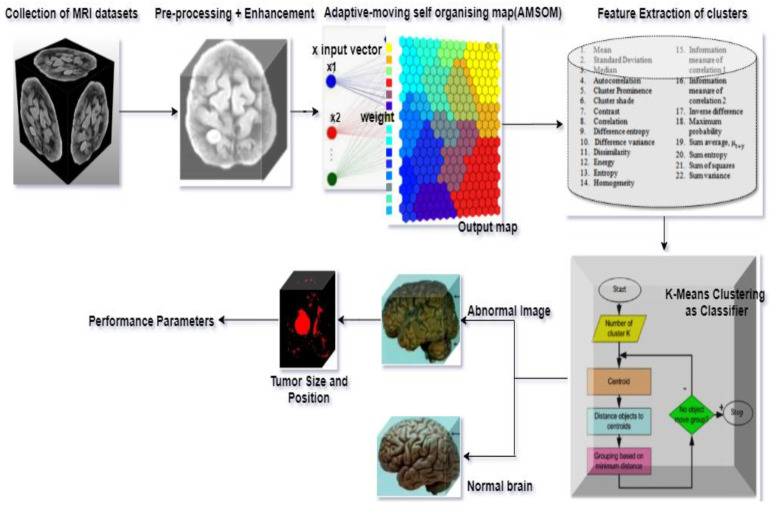
Process flow of the proposed method.

This research presents an efficient tumor detection and segmentation technique using an adaptive moving self-organizing map and Fuzzy K-mean clustering (AMSOM-FKM). The proposed approach mainly focused on segmenting tumors using extraction of the tumor region. The proposed method was implemented over MATLAB. The following phases were utilized. Pre-processing and development, function extraction, AMSOM segments, and K-means were achieved with efficient programming, as seen in Figure 4. Based on the algorithm, the image of the MRI was classified as usual and abnormal.

#### 3.2.1. Pre-Processing

The MRI image was pre-processed first and was then used for different processes. The input images were revamped to 256 × 256 pixels resolution during pre-processing without missing any image data. The essential tasks are to remove excessive image noise and remove patients’ names, ages, sex, place, address of residence, and skull. The representation of the RGB colour was then converted by 256 (0–255) into grey type. It was helpful to imagine the image quality and to achieve an ideal signal-to-noise level. Following the pre-processing steps, image enhancement was carried out [29].

#### 3.2.2. Image Enhancement

Numerous image processing methods were developed to increase image quality. Histogram equalization (EH) is one of the popular global image processes. That is the method of distributing the degree of prominence of the image over the full spectrum of histograms [30]. The suggested hybrid segmentation method was implemented from brightness to improve image quality during the next phase [31]. Suppose mb_i_ and mb_o_ are the mean brightness of the input image and the image function f obtained after equalization. In that case, grey pixel information is g-function obtained.

(1) $$g\left(l,m\right)=\frac{{\mathrm{mb}}_{i}}{{\mathrm{mb}}_{0}}f\left(l,m\right)$$

#### 3.2.3. Clustering

Adaptive moving self-organizing (AMSOM) is a unique clustering technique used by [32], which was used in this work for the segmentation of MRI. The first step begins with voxel intensities initially evaluated and modelled using SOM (self-organizing map) prototypes. It is unsupervised learning and groups the same form of characteristics into two or more dimensional lattices. At the same time, in the output space, different ones appear. The position vectors were initially determined in the same way as the positions of the neurons in the hexagonal grid structure, where the voxel intensities were initially evaluated and modelled using SOM prototypes. The input data were set at the input vector, and then RєI, rєR so $r\left(t\right).$ It was the input vector at time *t* and $s{\left(t\right)}_{i}^{1}$ is the raw vector at each input i. When the unit was closer to the winning neuron, it was defined as the best-winning neuron. It is calculated at each iterative step using [33].

(2) $$w_{s}\left(t\right)=\mathrm{argmin}\left\{\left\|r\left(t\right)-s{\left(t\right)}_{i}{}^{1}\right\|\right\}$$

The prototype was updated sequentially. The incremental process used the exponential decay learning factor and was the neighbourhood function.

(3) $$s_{i}\left(t+1\right)=s_{i}\left(t\right)+\mathrm{lr}\left(t\right)\cdot h_{\mathrm{Wi}}\left(t\right)\cdot [r\left(t\right)-s_{i}{\left(t\right)}^{1}]$$

Both factors are inversely proportional to time, so it decreases over time. The incremental process reduces as the neighbourhood weight factor falls, which is a few units. Here, it indicates the position of the exit space and $\|n_{u}-n_{i}\|\;$ is the distance between the winning unit and the space. The Euclidean distance is computed as $\left\|r\left(t\right)-s{\left(t\right)}_{i}{}^{2}\right\|$. However, after the initial process, the orthogonal and symmetric matrices *T* and *P* of the same size, where T (p, q) means 0 for no relation, 1 is a connected neuron, and P (p, q) indicates the boundary age of the neurons [21]. The neurons are nearest neighbours in the present era, meaning there is 0. Yet, another value means neurons are the closest neighbours. The *MTr* threshold is a function of the data dimension (*D*) given by the MTr threshold [34,35,36].

(4) $$\mathrm{MTr}\;=-\ln\left(-D\right)\times \ln\left(F\right)$$

In adaptive learning, the neurons’ weight is approximated by an algorithm of the SO’M array, where wi (t + 1) is used as

(5) $$\mathrm{wi}\left(t+1\right)=\frac{\sum_{i}^{i=1}\;\mathrm{nj}\left(t\right)\times \exp\left(-\frac{\|\mathrm{rj}-\mathrm{ri}{\|}^{2}}{2\sigma {\left(t\right)}^{2}}\right)\times \mathrm{xej}\left(t\right)}{\sum_{i}^{j=1}\;\mathrm{nj}\left(t\right)\times \exp\left(-\frac{\|\mathrm{rj}-\mathrm{ri}{\|}^{2}}{2\sigma {\left(t\right)}^{2}}\right)}$$

where *nj(t)* is an integer, *hji(t)* is the neighborhood function, *xej(t)* is the average vector of x, and *xej(t)* is an adaptive feature. The distances between the neuronal vectors (*wi*) are determined at each point and after modifying the neuronal weight vectors. These distances measure neuronal similarity in the input region [34].

(6) $$\mathrm{ri}\left(t+1\right)=\mathrm{ri}\left(t\right)+\left(0.01\right)\frac{\sum_{j}^{i=1}\;\mathrm{nj}\left(t\right).\delta \mathrm{ji}\left(t\right).(\mathrm{rj}\left(t\right)-\mathrm{ri}\left(t\right)}{\sum_{j}^{i=1}\;\mathrm{nj}\left(t\right).\delta \mathrm{ji}\left(t\right)}$$

Value 0.01 explains *δji(t)* is a distinct feature, *μ* is a neighbourhood function, *γ* controls the diminishing district as a fraction. Since learning is complete, no neurons are added or deleted. However, at a lower rate, weight and location adaptation vectors are continued [37]. With the following equation, the cluster is produced:

(7) $$\mathrm{cluster}=\overset{k}{\underset{i=0}{\sum }}\;\overset{n}{\underset{i=0}{\sum }}{\;u}_{\mathrm{ij}}\times \exp\left(-\frac{\|\mathrm{wj}-\mathrm{wi}{\|}^{2}}{\gamma \times \sigma {\left(t\right)}^{2}}\right)$$

Here, the membership element is. “$u_{\mathrm{ij}}$”. The number of AMSOM clusters is “k”, and the number of input pixels is identified as “n”.

#### 3.2.4. Feature Extraction

In this function, the clusters generated were considered for the extraction feature. The matrix was constructed at a distance of d = 1 and angles $\left(\theta \right)$ of degrees (0, 45, 90, 135). It is calculated at different angles. Grey-level co-occurrence matrix (GLCM) is a textured character. This profile refers to the touch, i.e., smooth, silky, rough, etc [38,39,40,41,42,43].

**Figure 5:**
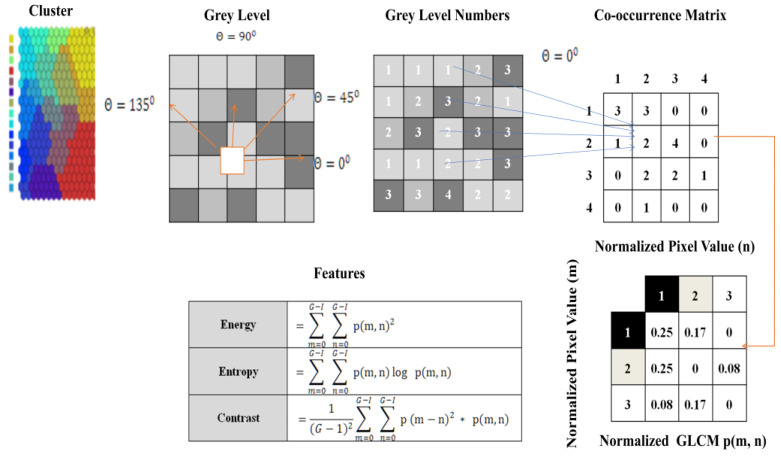
Process of feature extraction using AMSOM cluster.

**Figure 6:**
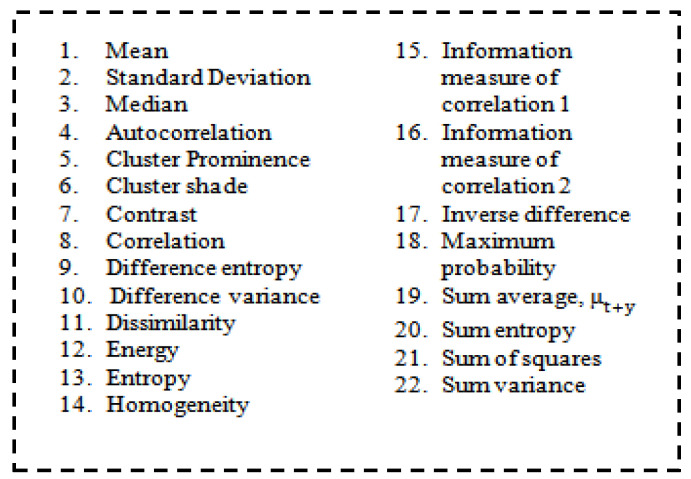
List of features.

The order of statics was as follows: first-order texture steps were statistics reported from the original image values, such as variance, and pixel neighbour relationships were not implemented. Second-order measures describe the relationship between groups of two (usually adjacent) pixels in the original image. GLCM is the feature extraction tool used to analyze the textures considered for examining feature analysis [44]. Figure 5 shows how the grey-level co-occurrence matrix features differentiated the surface of an image by measuring how often pixel pairs of different values and in a given spatial $\;\left(\theta \right)$ relationship occurred in a photo [45], generating a matrix and then extracting the statistical measures described in Figure 6.

### 3.3. Classification

FKM assigned each object to its group and calculated the distances between all the groups based on the chosen linking criteria to merge the two closest until only one cluster remained. The estimated distance was produced using [46] Equation (8).

(8) $$Y_{k}=\overset{M}{\underset{p=1}{\sum }}\;\overset{N}{\underset{q=1}{\sum }}\;[d_{\mathrm{pq}}^{\frac{1}{m-1}}\overset{k}{\underset{i=1}{\sum }}\;{\frac{1}{d_{\mathrm{pq}}}}^{\frac{1}{m-1}}{]}^{-1}d_{\mathrm{pq}}$$

where k is no. of iteration, N is the data points or pixels present in the input image, M is the number of clusters formed by FKM, m is the fuzziness coefficient, $d_{\mathrm{pq}}$ is Squared Euclidean distance between pixel xi calculated as $\left\|R\left(p-c_{j}\right)\right\|$ the co-efficiently obtained due to the overlapping of clusters.

(9) $$\mathrm{Clc}=\frac{\sum_{p=1}^{N}\;\cup_{p,q}^{m}I\left(p\right)}{\sum_{p=1}^{N}\;\cup_{p,q}^{m}}$$

### 3.4. Volume Estimation

It was evaluated using connected region calculation on segmented images by assessing the number of pixels covered over the total number of pixels in mm^3^.

### 3.5. Performance Parameters

The significant quality parameters that determine the precision and efficiency of the proposed algorithm are presented as statistical measures, such as the mean square error, and others were evaluated using Equations (10)–(19). These statistical measures were briefly defined in [47,48,49,50,51,52,53].

(10) $$\mathrm{MeanSquareError}=\frac{1}{m}\overset{m=1}{\underset{i=0}{\sum }}\;\overset{n=1}{\underset{j=0}{\sum }}\;\left[R\left(i,j\right)-S\left(i,j\right)\right]$$

(11) $$\;\mathrm{PSNR}=10\log\frac{256\times 256}{\mathrm{MeanSquareError}}\mathrm{dB}$$

Computational time =Pre-Processing In Segmentation process+ Classification Process

(12) $$\begin{array}{cc} \mathrm{Computational}\;\mathrm{time} & \\ = & Pre-Processing\_In\_Segmentation\_process\\ + & Classification\;Process \end{array}$$

(13) $$\mathrm{TanimotoCoefficient}=\frac{s\left(R\cap S\right)}{s\left(R\cup S\right)}$$

(14) $$\mathrm{DiceCoefficientIndex}=\frac{\left(2\times \mathrm{TanimotoCoefficient}\right)}{\left(1+\mathrm{TanimotoCoefficient}\right)}$$

(15) $$\mathrm{SimilarityIndex}\;=\frac{1}{1+\frac{\mathrm{FP}+\mathrm{FN}}{2\times \mathrm{TP}}}$$

(16) $$\mathrm{OverlapFraction}\;=\frac{1}{1+\frac{\mathrm{FN}}{\mathrm{TP}}}\times 100\%\;$$

(17) $$\mathrm{Accuracy}=\frac{\left(\mathrm{TP}\;+\;\mathrm{TN}\right)}{\left(\mathrm{TP}\;+\;\mathrm{TN}\;+\;\mathrm{FP}\;+\;\mathrm{FN}\right)}\times 100\%\;$$

(18) $$\mathrm{Sensitivity}=\frac{\left(\mathrm{TN}\right)}{\left(\mathrm{TP}\;+\;\mathrm{FN}\right)}\times 100$$

(19) VolumeEstimation = Pixelsize×Extracted_region

where R is a raw image, S is segmented, TP is a true positive, TN is a true negative, FP is a false positive, and FN is a false negative [53].

### 3.6. Proposed AMSOM-FKM Algorithm

The steps of the proposed Algorithm 1 are as follows.
**Algorithm 1** Proposed AMSOM-FKM AlgorithmInput: MRI image dataset Output: Tumor and non-tumor imagesStep 1.Convert the colour image to grayscale and resize it to 256 × 256-pixel data.Step 2.Compute pre-processing of data using histogram equalization. It modifies the brightness of images to improve contrast.Step 3.Compute image segmentation and extraction of the region of interest using adaptive moving self-organizing and decide on the AMSOM’s starting grid layout and size.Step 4.Initialize the structure and size of a rectangular grid with several neurons N and initialize vector position.Step 5.Calculate the moving threshold by the dimension of the data generated using the GLCM matrix and find the winning neurons.Step 6.Compute the centroid points for the following using the Euclidean Distance metric.Step 7.Centroid points are fed to cluster the data and produce K-means clusteringStep 8.Compute the size of the tumor in MR Image. With the help of the size of the tumor, it classifies tumor and non-tumor images.

## 4. Result and Analysis

**Figure 7:**
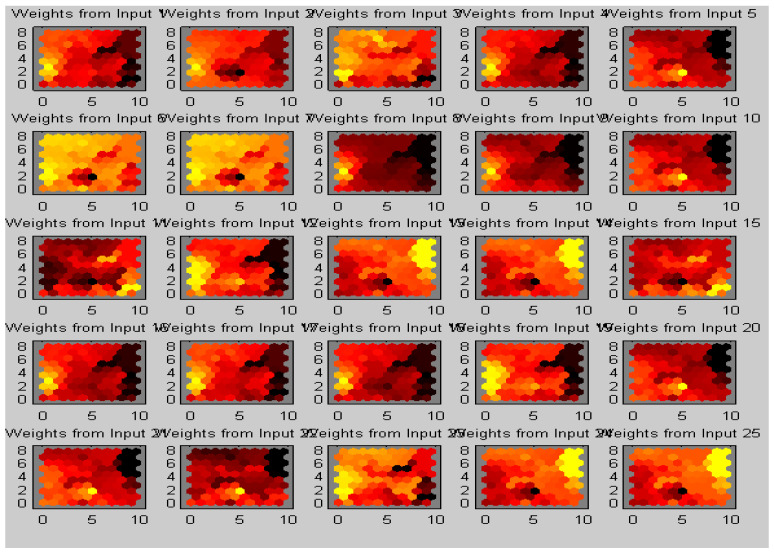
AMSOMV diagram illustrates 5 × 5 maps with varying colours as clusters in each map.

**Figure 8:**
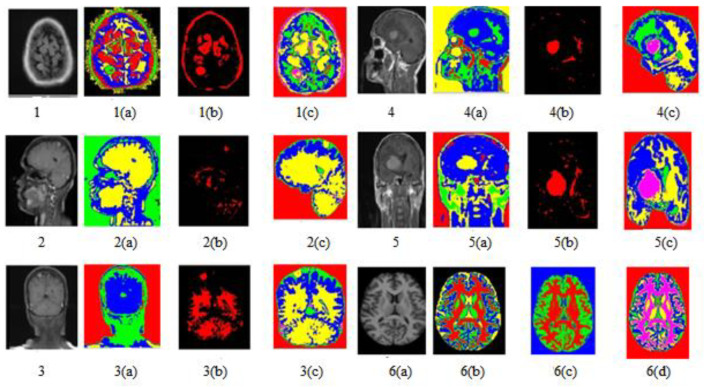
Image segmentation of T1, T2, and FLAIR image sequences.

The work was carried out on a laptop with an Intel (R) Core (TM) i5-5005U CPU @ 2.00 GHz with 8 GB of RAM using the MATLAB software version (R2018b). The results of our proposed algorithm were obtained from 42 real data sets of MRI images of different age groups and genders that predict the tumor’s type, position, and area. The pre-processing was performed with the removal of the skull and image enhancement followed by clustering, whose Voronoi output is shown in Figure 7. The images were pooled for image segmentation and comparing the algorithm shown in Figure 8. This table calculated the validation parameters that offer a low mean square value concerning the previous algorithm, such as KMFCM and SOM-FKM.

Figure 8 shows all abnormal images. Figure 8(1) is the axial flair image with meningioma; Figure 8(2) is T1-Sagittal obtained from the 35-year-old patient suffering from PNET (primitive neuroectodermal tumor); Figure 8(3) is T1 Coronary with Contrast Enhancement, Figure 8(4) and Figure 8(5) show unclear identification of the Tumor region and portions of edema.

Figure 8(6) is a standard brain image. The lateral ventricular system GM and WM were not identified using the SOM-FKM in Figure 8(1a,1b) KMFKM algorithm. However, perfect tissue separation and tumor identification was performed with the proposed methodology AMSOM-FKM in Figure 8(1c). The result produced with SOM-FKM cannot identify the Tumor, so AMSOM placed a transparent Tumor region Figure 8(4a,5a) show unclear identification of the Tumor region portions edema with SOM-FKM, KMFCM.

However, the proposed algorithm produced a clear and good tissue group with a separate area of tumors and edema shown in Figure 8(4c) in the result with AMSOM-FKM was distinguished are available and seen in result Figure 8(1c). Peak signal noise ratio (PSNR) and mean square error (MSE) compare the squared error between the original and the reconstructed image. There is an inverse relationship between PSNR and MSE so, so a higher PSNR value indicates a higher image quality (better). The groups produced and the validation parameters show that MSE and PSNR were 0.03 and 62.91 dB, satisfying the algorithm’s efficiency.

**Figure 9:**
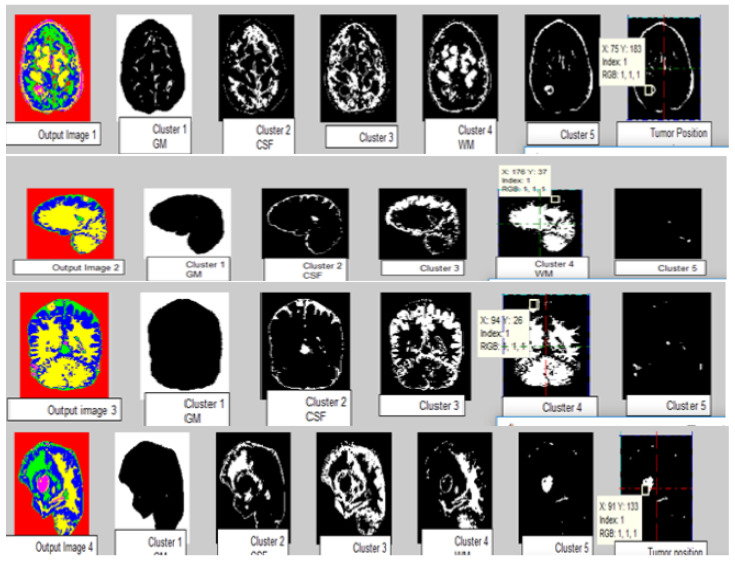
Tumor size and location using Output segmented images. Output Image 1 produces 5 clusters with the exact Tumor in cluster 5 with positions X = 75 mm, Y = 183 mm. Output Image 2 has 5 clusters with positions X = 176 mm and Y = 37 mm. Output Image 3 with position X = 94 mm, Y = 26 mm. Output Image 4 produces 5 clusters with positions X = 99 mm and Y = 133 mm.

The images of different clusters formed during the clustering extraction of the tumor region are clearly illustrated in Figure 9. Perfect tumor identification and tissue segmentation with other sets for size estimation are shown in Figure 9. Output Image 1 produced five clusters with an exact tumor in cluster 5 with positions X = 75 mm and Y = 183 mm. Output Image 2 had five clusters with positions X = 176 mm and Y = 37 mm. Output Image 3 with position X = 94 mm, Y = 26 mm. Output Image 4 had five clusters with positions X = 99 mm and Y = 133 mm.

There was a high variation in tumor size, directly correlating with the disease status and medical approach. If such minute-size tumors can be identified, it will reduce misdiagnosis and enhance early diagnosis. Based on the datasets, machine learning algorithms were finalized for the model. Underfitting and overfitting are the two main conditions that can affect accuracy. Underfitting occurs when data are less, and overfitting occurs when data are extensive. So, an excellent fit algorithm must be used for better performance. Table 2 shows the experimental results of the proposed AMSOM-FKM and existing methods, i.e., KMFCM, SOM-FKM, and AMSOM [54].

Table 2 illustrates the size of extracted tumor and edema region. The comparative analysis with methodology perceptive and accuracy evaluated was compared and is shown in Table 3. The 22 features with AMSOM-FKM used by the proposed algorithm were the overcoming factor for all other techniques. The average accuracy of KMFCM [20] was 98%, SOM-FKM [19] was 94%, and the proposed was high at 99.8%.

The MRI images of different data compared with different algorithms implemented by researchers formed in one tumor region are clearly illustrated in Table 4. Tumor identification and tissue segmentation with other methods enhanced efficiency and accuracy as the OTSU method had a high accuracy of 97.3%. In contrast, the hybrid-clustering technique had 97.69% accuracy. Table 4 clearly states that the accuracy was improved as the features increased, so the proposed method considered 22 features.

**Figure 10:**
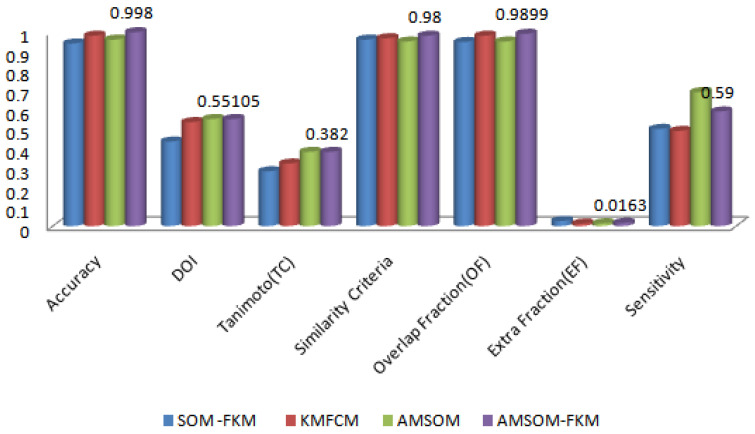
Comparative analysis of various algorithms over validation parameters.

As is clear from Figure 10, the different validating parameter is represented on the figure’s *x*-axis, and corresponding values are present on the *y*-axis, which has no unit. Accuracy, DOI, Tanimoto index, similarity criteria, overlap fraction and extra fraction, and sensitivity were the parameters compared. As the accuracy was high, the algorithm was efficient. As the sensitivity was high, the algorithm was sensitive to variation or noise. This method showed that the proposed algorithm had better accuracy than existing techniques.

**Figure 11:**
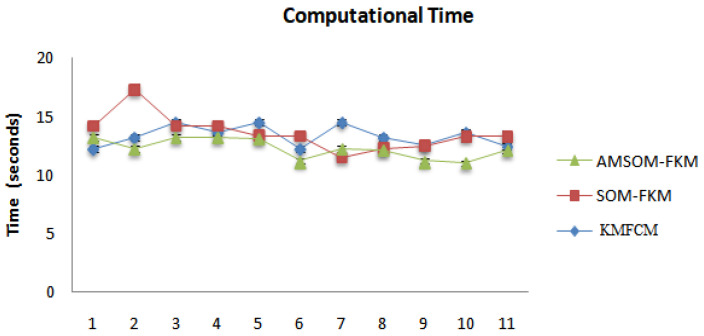
Comparative analysis of various algorithms for time consumption.

The proposed algorithm allowed neurons to change positions during training, providing better visualization and faster training time, as shown in Figure 11. Therefore, cluster numbers were adequate and accurate cluster points that adequately segmented tissue regions. The proposed algorithm also ranked higher in producing better DOI and TC values. The average DOI and TC values produced by the AMSOM-FKM algorithm were 0.435105 and 0.282381. The accuracy shown in Table 4 revealed that the proposed algorithm produced satisfactory results with 99.8% accuracy. Table 5 demonstrates the performance evaluation regarding the Dice score and Jaccard index below.

**Figure 12:**
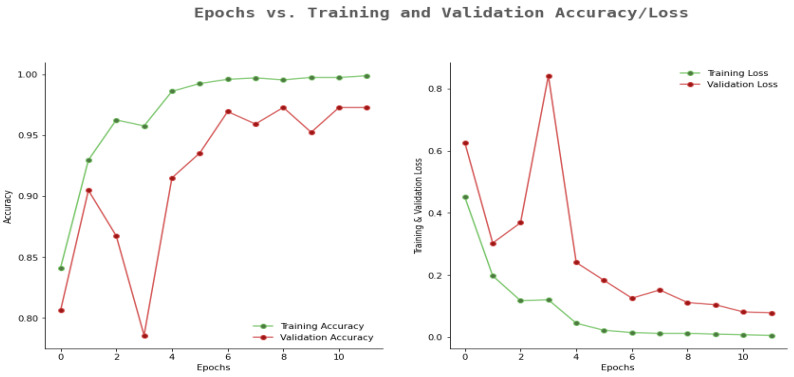
Proposed model accuracy and loss results.

Similarly, Figure 12 shows the outcomes of the proposed model in terms of epoch and accuracy/loss results. The proposed model had better training accuracy and validation for more epochs. Additionally, we observed that training and validation loss were more elevated once the number of epochs was less. However, when the number of epochs increased, the training and validation loss was less.

## 5. Conclusions and Future Work

In this research, we developed an efficient brain tumor segmentation method AMSOM-FKM based on an adaptive moving self-organizing map and the Fuzzy K-mean clustering method. Specifically, the Brats18 MRI Tumor Image database was used for this study. Detecting and extracting the heterogeneous tumor area from the many MR brain images in the collection is difficult. The suggested method demonstrated superior performance over the AMSOM and FKM algorithms for solving the segmentation and segregation issues in the tumor area. By integrating prior information with characteristics extracted from brain MR images, classifiers may be created for the segmentation techniques. The proposed method and existing KMFCM, SOM-FKM, andAMSOM were generated using MATLAB and various performance measuring parameters, i.e., detection rate, accuracy, loss validation, MSE, PSNR, and DOI. The proposed method achieved more than 10% better results than existing methods.

In future work, the proposed methodology can be used in radiology for the everlasting detection and position of the tumor. The same methods can also classify and analyze pathologies like Parkinson’s disease. The suggested soft computing algorithms should be used in the field programmable gate array (FPGA) of a clinical MRI scanner so that the regions and tissues found in the brain can be easily visualized.

## Figures and Tables

**Table 1:** Related works summary.

References	Model	Dataset	Feature Validation	Performance Remarks
[1]	ResNet50, VGG19, InceptionV3, MobileNetand Class Activation Maps (CAMs)	3441 MRI images	No	96.45% with ResNet50, 93.40% with VGG19, 85.03% with InceptionV3 and 89.34% with MobileNet
[2]	DarkNet model	T1W-CE MRI dataset	No	98.84% Accuracy
[3]	Convolution neural network and long short-term memory	1000 MRI images dataset	No	97.5% Accuracy
[4]	Adaptive Neuro-Fuzzy Inference System and Support Vector Machine	MRI images dataset	No	85.74% Accuracy
[5]	U-Net model	BRATS dataset	No	89% Accuracy
[6]	ResNet50 network	Cancer Genome Atlas Low-Grade Glioma (TCGA-LGG) database	No	92.34% Accuracy
[7]	nnU-Net	ICTS dataset	No	87.23% Accuracy
[8]	Discrete Cosine Transform (D.C.T.), CNN, and ResNet50	ToloharbourDataset	No	98.14% Accuracy
[9]	Coupling real-time intraoperative imaging modalities	TumorID endogenous fluorescence imaging system	No	1.45 RMSE (Root-Mean-Square Error)
[10]	VGG Stacked Classifier Network	253 MRI ImagesKaggle	No	99.2% Accuracy
[11]	Convolutional neural network	GBM data set	No	98% Accuracy
[12]	Inception-v3 and DensNet201	3064, T1-weighted contrast MR images	No	99.34%, and 99.51% with Inception-v3 and DensNet201
[13]	Deep neural networks (DNN.)	RIDER (Reference Image Database)	No	0.93 ± 0.14 Accuracy
[14]	Kernel support vector machine (KSVM)	306 brain images by Shengjing Hospital of China Medical University	No	97.83% Accuracy
[15]	Convolution neural network	MICCAI BraTS 2018	No	0.995 sensitivity (SN) and 0.997 specificities (SE.)
[16]	Template-based K means, and Fuzzy C means	MRI images	No	97.5% Accuracy
Proposed Model	AMSOM-FKM	1691 images from BraTS 2018 dataset	Yes	Higher precision, Recall. Better training accuracy and less Validation loss.

**Table 2:** Experimental results (Proposed vs. Existing Method).

Algorithm	MSE	PSNR	DOI	TC
KMFCM	0.07	59.45	0.3	0.24
SOM-FKM	0.07	59.70	0.33	0.22
AMSOM	0.1	58.14	0.34	0.2
**AMSOM-FKM (Proposed)**	**0.03**	**62.91**	**0.39**	**0.24**
KMFCM	0.08	58.84	0.5	0.34
SOM-FKM	0.09	58.35	0.33	0.22
AMSOM	0.1	55.42	0.38	0.23
**AMSOM-FKM (Proposed)**	**0.02**	**63.42**	**0.53**	**0.36**
KMFCM	0.1	57.6	0.50	0.33
SOM-FKM	0.13	67.16	0.62	0.45
AMSOM	0.09	58.66	0.34	0.20
**AMSOM-FKM (Proposed)**	**0.04**	**61.93**	**0.47**	**0.31**
KMFCM	0.08	59.12	0.50	0.36
SOM-FKM	0.1	66.92	0.40	0.22
AMSOM	0.08	58.2	0.33	0.20
**AMSOM-FKM (Proposed)**	**0.02**	**63.68**	**0.53**	**0.36**
KMFCM	0.07	59.35	0.48	0.32
SOM-FKM	0.66	54.8	0.38	0.23
AMSOM	0.07	59.88	0.33	0.20
**AMSOM-FKM (Proposed)**	**0.03**	**63.25**	**0.48**	**0.32**
KMFCM	0.12	57.24	0.67	0.45
SOM-FKM	0.05	59.48	0.85	0.43
AMSOM	0.17	40.48	0.01	0.201
**AMSOM-FKM (Proposed)**	**0.037**	**60.48**	**0.401**	**0.3014**

**Table 3:** Estimation of tumor size.

Estimated Size	Actual Volume	Difference	% Error
Tumor region = 302, pixel size = 1 mm, Tumor size = 302 mm^3^	219 mm^3^	83 mm^3^	1.4
Tumor region = 288, pixel size = 1 mm, Tumor size = 288 mm^3^	284 mm^3^	4 mm^3^	1.0
Tumor region = 387, pixel size = 1 mm, Tumor size = 387 mm^3^	322 mm^3^	65 mm^3^	1.2
Tumor region = 615, pixel size = 1 mm, Tumor size = 615 mm^3^, Edema region = 3522, Edema size = 3522 mm^3^	601 mm^3^	14 mm^3^	1.1

**Table 4:** Accuracy analysis with various techniques.

Techniques	Filters	Features	Segmentation	Classification	Accuracy (Average) (%)
SOM–FKM [18]	Median	4 features	SOM-FKM	-	94
AMSOM [19]	Median	3 features	AMSOM	-	96
KMFCM [20]	BCDHE	AGLCM 9 features	KMFCM	SVM	98
CNN [24]	Median	Intensity	Learning without Forgetting (LwF)	Bayesian Optimization	84.52
Hybrid clustering [23]	Genetic Median Filter	GLCM and Gabor feature	Hierarchical Fuzzy clustering	Lion Optimization BSVM	97.69
CNN [38]	SLIC	Momentum	LeakyReLU	Bayesian Optimization	98.3
AMSOM-FKM (Proposed)	BCDHE	GLCM 22 features	AMSOM	FKM	99.8

**Table 5:** Dice score and Jaccard Index analysis with various techniques.

S. No.	Authors	Dice Score	Jaccard Index
1	CNN [24]	0.717	72.56
2	Hybrid Clustering [23]	0.791	89.14
3	KMFCM [20]	0.896	68.14
4	CNN [38]	0.918	95.31
**5**	**AMSOM-FKM (Proposed)**	**0.956**	**98.25**

## Data Availability

The dataset will be available with the corresponding author based on individual requests.
